# The complete chloroplast genome sequence of *Mikania micrantha* (Asteraceae), a noxious invasive weed to South China

**DOI:** 10.1080/23802359.2016.1209090

**Published:** 2016-08-31

**Authors:** Lu Huang, Zhen Wang, Ting Wang, Ying-Juan Su

**Affiliations:** a School of Life Sciences, Sun Yat-Sen University, Guangzhou, China;; b College of Life Sciences, Nanjing Agricultural University, Nanjing, China;; c College of Life Sciences, South China Agricultural University, Guangzhou, China;; d Research Institute of Sun Yat-Sen University in Shenzhen, Nanshan District, Shenzhen, China

**Keywords:** Asteraceae, chloroplast genome, *Mikania micrantha*, noxious invasive weed

## Abstract

It is the first report on complete chloroplast genome of *Mikania micrantha* (Asteraceae), a noxious invasive weed to South China. The genome is a circular molecule of 152,092 bp in length with 37.58% average GC content, and includes a large single copy region (83,793 bp), a small single copy region (18,287 bp), and two inverted repeat regions (25,006 bp). The *M. micrantha* cp genome encodes 135 unique genes, including 90 protein-coding genes, 37 tRNA genes, and 8 rRNA genes. ML tree based on 16 complete cpDNA from Asteraceae indicated that *M. micrantha* has a close sister relationship with *Ageratina adenophora* and *Praxelis clematidea*. The complete cpDNA of *M. micrantha* provides useful molecular data for further phylogenetic and evolutionary analysis.

*Mikania micrantha* (Asteraceae) is one of the top 10 worst weeds in the world known as a mile-a-minute weed. It is an extremely fast growing, long-lived herbaceous vine. It was introduced to Hong Kong in 1884 (Zhang et al. 2004). After naturalization, *M. micrantha* has rapidly spread through the Pearl River Delta since 1984, due to lack of natural enemy (Huang et al. 2015). The species is a noxious invasive weed to South China. It not only kills other plants by blocking the light, twinning, smothering and preventing forest tree regeneration (Zhang et al. 2004), but also competes for water and nutrients with coexisting natives (Wang et al. 2004). Currently, studies on *M. micrantha* focus in investigation of basic features such as the biological characteristics (Hu & But 1994), photosynthetic activity (Wen et al. 2000), allelopathy (Shao et al. 2003), and genetic variation (Wang et al. 2008). However, its characteristics of chloroplast genome remain unresolved. Here, we reported the complete cp genome sequences of *M. micrantha*, which will contribute to elucidate adaptive evolutionary mechanism.

The leaves were from bamboo garden (N: 23°5'37.41”, E: 113°17'43.94”), Sun Yat-sen University, Guangdong Province, China, and the specimen is stored in Herbarium of Sun Yat-sen University (SYSU; voucher: *L Huang 201408*). We sequenced the complete cp genome by using Illumina HiSeqTM 2500 platform (Illumina Inc., San Diego, CA), which generated a total of 4.73 GB of 150-bp paired-end clean reads. Velvet (V 1.2.07) was used to assemble *de novo* sequences from clean reads (Zerbino & Birney 2008). A total length of 127,801 bp in ten chloroplast contigs were obtained through BLAST to the published invasive species cpDNA sequences in Asteraceae. PCR amplification was applied to bridge the gaps. The initial annotation of the assembled chloroplast genome was executed with Dual Organellar GenoMe Annotator (DOGMA) to predict protein coding genes, transfer RNA (tRNA) genes, and ribosome RNA (rRNA) genes. Furthermore, we performed BLASTN to determine accurate annotation of gene positions. Based on complete chloroplast sequences of 15 species corresponding to 15 different genera in Asteraceae, a maximum likelihood tree was constructed to explore phylogenetic relationship of *M. micrantha* using RAxMLGUI v.1.3 with 1000 bootstrap replicates (Silvestro & Michalak 2012). *Trachelium caeruleum* (Campanulaceae) was used as outgroup.

The complete cp genome of *M. micrantha* is a circular molecule of 152,092 bp in length with 37.58% average GC content (GenBank accession no. KX154571), and includes a large single copy region (LSC, 83,793 bp), a small single copy region (SSC, 18,287 bp), and two inverted repeat regions (IR, 25,006 bp). The *M. micrantha* cp genome contains 135 unique genes, including 90 protein-coding genes, 37 tRNA genes, and 8 rRNA genes. Among these genes, two (*clpP* and *ycf3*) contained two introns, which were located in LSC. In addition, 19 genes embraced one introns. ML result with 98% bootstrap showed that *M. micrantha* has a close sister relationship with *Ageratina adenophora* and *Praxelis clematidea* (Figure 1). The complete cpDNA of *M. micrantha* provides useful molecular data for further phylogenetic and evolutionary analysis.

**Figure 1.**
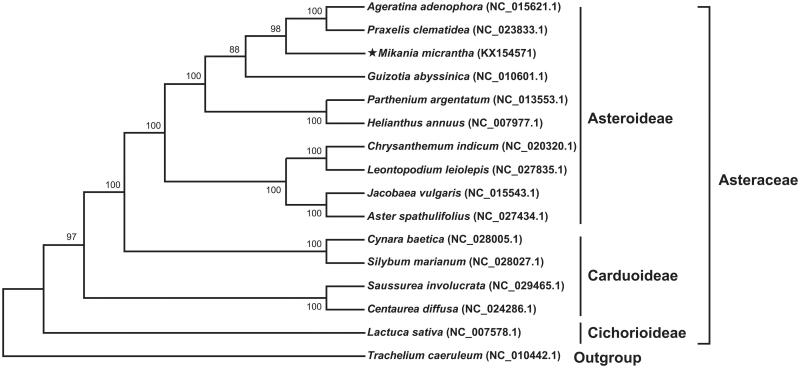
The ML phylogenetic tree based on complete chloroplast genome sequences of 15 species in Asteraceae and *Trachelium caeruleum* as outgroup. The complete chloroplast genome sequences were downloaded from GenBank, and the accession numbers were shown in the tree. ML bootstrap support values were indicated at the nodes.

## References

[CIT0001] Hu YJ, But PPH. 1994 A study on life cycle and response to herbicides of *Mikania micrantha.* Acta Sci Nat Univ Sunyatseni. 33:88–95. (in Chinese).

[CIT0002] Huang QQ, Shen YD, Li XX, Zhang GL, Huang DD, Fan ZW. 2015 Regeneration capacity of the small clonal fragments of the invasive *Mikania micrantha* H.B.K: effects of the stolon thickness, internode length and presence of leaves. Weed Biol Manag. 15:70–77.

[CIT0003] Shao H, Peng SL, Zhang C, Xiang YC, Nan P. 2003 Allelopathic potential of *Mikania micrantha.* Chin J Ecol. 22:62–65. (in Chinese).

[CIT0004] Silvestro D, Michalak I. 2012 RaxmlGUI: a graphical front-end for RAxML. Org Divers Evol. 12:335–337.

[CIT0005] Wang T, Su YJ, Chen GP. 2008 Population genetic variation and structure of the invasive weed *Mikania micrantha* in southern China: consequences of rapid range expansion. J Hered. 99:22–33. 17906304 10.1093/jhered/esm080

[CIT0006] Wang BS, Wang YJ, Liao WB, Zan QJ, Li MG, Peng SL, Han AC, Zhang WY, Chen DP. 2004 The invasion ecology and management of alien weed Mikania micrantha H.B.K. Beijing: Science Press pp. 152–177.

[CIT0007] Wen DZ, Ye WH, Feng HL, Cai CX. 2000 Comparison of basic photosynthetic characteristics between exotic invader weed *Mikania micrantha* and its companion species. J Tropi Substropi Bot. 8:139–146. (in Chinese).

[CIT0008] Zerbino DR, Birney E. 2008 Velvet: algorithms for de novo short read assembly using de Bruijn graphs. Genome Res. 18:821–829. 18349386 10.1101/gr.074492.107 PMC2336801

[CIT0009] Zhang LY, Ye WH, Cao HL, Feng HL. 2004 *Mikania micrantha* H.B.K. in China-an overview. Weed Res. 44:42–49.

